# Quercetagetin alleviates zearalenone-induced liver injury in rabbits through Keap1/Nrf2/ARE signaling pathway

**DOI:** 10.3389/fphar.2023.1271384

**Published:** 2023-10-03

**Authors:** Fengyang Wu, Fengxia Wang, Zhaohong Tang, Xinyu Yang, Yanhua Liu, Man Zhao, Shudong Liu, Shuaijuan Han, Zhisheng Zhang, Baojiang Chen

**Affiliations:** ^1^ College of Animal Science and Technology, Hebei Agricultural University, Baoding, China; ^2^ College of Food Science and Technology, Hebei Agricultural University, Baoding, China; ^3^ Hebei Research Institute of Microbiology Co., Ltd., Baoding, China

**Keywords:** Quercetagetin, Zearalenone, rabbit, liver injury, Keap1/Nrf2/ARE signaling pathway

## Abstract

**Introduction:** This study aimed to assess the alleviative effect of quercetagetin (QG) on zearalenone (ZEN)-induced liver injury in rabbits.

**Methods:** Ninety 41-day-old healthy Hyla rabbits were randomly assigned into three groups, including a control (fed with basic diet), ZEN addition group (fed with basic diet + 600 μg/kg ZEN), and ZEN + QG addition group (fed with basic diet + 600 μg/kg ZEN + 100 mg/kg QG), with 30 rabbits per group. The duration of the experiment was 28 days.

**Results:** The results revealed no significant differences in the average daily gain, average daily feed intake, the gain to feed ratio and the liver, kidney and spleen organ indexes (*p* > 0.05) between the rabbits across the three groups. However, the sacculus rotundus index of the rabbits in the control group was significantly higher than that in the ZEN + QG group (*p* < 0.05). The intake of ZEN-contaminated diet also significantly increased the activities or levels of alanine transaminase, alkaline phosphatase, total bile acid (TBA), total bilirubin, malondialdehyde, and interleukin-4 (IL-4) and enhanced the abundance of kelch-like ECH-associated protein 1 *(Keap1)*, heat shock protein 70 *(HSP70)* and cysteine-aspartic acid protease-3 *(Caspase-3)* mRNA in the blood or liver tissue in ZEN group, compared to the control group (*p* < 0.05). On the contrary, the activities or levels of immunoglobulin A, complement 3, total antioxidant capacity, glutathione peroxidase (GSH-Px), superoxide dismutase, interleukin-10, and the abundance of nuclear factor E2-related factor 2 *(Nrf2)* and heme oxygenase-1 *(HO-1)* mRNA were significantly decreased (*p* < 0.05). Supplementing the diet with QG still maintained significantly higher levels of TBA and IL-4, and the abundance of *GSH-Px, HSP70, IL-4,* and *Caspase-3* mRNA in the blood and liver of rabbits in the ZEN + QG group than in the control group (*p* < 0.05). At the same time, the other indicators were restored to levels in the control group (*p* > 0.05).

**Discussion:** In conclusion, QG alleviated the ZEN-induced oxidative damage and liver injury caused by inflammatory reaction through the Keap1-Nrf2-antioxidant response element (ARE) signal pathway, which protected the liver. This study revealed the alleviative effect of QG on the hepatotoxicity of ZEN in rabbits for the first time, providing a new perspective for applying QG and developing a ZEN antidote.

## 1 Introduction

Zearalenone (ZEN) is a 2,4-dihydroxybenzoic acid lactone compound produced by various *Fusarium* species (Urry et al., 1966). It widely exists in the crops and food products. ZEN pollution adversely affects the food appearance, smell, and nutritional value. It induces toxic effects, including reproductive liver, kidney, immune, digestive tract, and genetic toxicities, harmful to rabbits at all growth stages. Therefore, ZEN is one of the most common and harmful mycotoxins in rabbit production (Conková et al., 2001; Li et al., 2018; Tsouloufi et al., 2018; Tsouloufi et al., 2019). The liver is an important organ for metabolism and detoxification. In rabbits, it is also the main organ for ZEN metabolism and transformation (Kuiper-Goodman et al., 1987). However, ZEN reduces the antioxidant capacity of the liver, leading to inflammation and liver injury of piglets (Wu et al., 2022a). In mouse liver, ZEN causes oxidative damage and inflammatory reaction, increasing hepatocyte apoptosis rate and liver injury (Wu et al., 2023). ZEN is also hepatotoxic to rabbits, which significantly increases the alanine transaminase (ALT), aspartate aminotransferase (AST), alkaline phosphatase (ALP), *γ*-glutamyltransferase and total lactate dehydrogenase activities in rabbits (Conková et al., 2001). However, there are few studies on alleviating ZEN-induced liver injury in rabbits.

Quercetagetin (QG), chemically identified as 3,3,4,5,6,7-hexahydroxyflavone, is a flavonol compound extracted from marigold (Asteraceae family) (Xu et al., 2011). It is a polyhydroxyphenol compound with hydrogen donor substituents on the benzene ring, which have a strong capacity to scavenge 2,2-biphenyl-1-picrylhydrazinyl (DPPH), hydroxyl radicals (·OH), 2,2′-diazobis (3-ethylbenzothiazoline-6-sulfonic acid) diamine salt (ABTS) and other free radicals, thereby serving as a natural antioxidant (Fuentes et al., 2021). QG increases the antioxidant enzymatic activities in the liver, ileum, and jejunum of broilers and reduces the level of malondialdehyde (MDA) through the nuclear factor E2-related factor 2 (Nrf2)/antioxidant response element (ARE) signal pathway mediated by Kelch-like ECH-associated protein 1 (Keap1) to improve the broiler antioxidant performance, intestinal structure, and morphology, and promote the nutrient digestibility (Wu et al., 2022b). QG also has immunomodulatory and anti-inflammatory effects. For example, QG inhibits the production of macrophage-derived chemokine by human keratinocytes. As a result, it is used as an immunotherapeutic agent in treating atopic dermatitis (Rufino et al., 2021). Besides, QG has biological functions such as anti-virus, anti-diabetes, and anti-hyperlipidemia (Seyedi et al., 2016; Wang et al., 2016).

It is unclear whether QG can alleviate the effect of ZEN on rabbit liver. Thus, in this study, QG was added to the ZEN-contaminated diet of rabbits to investigate its effect on the liver, providing a reference for applying QG.

## 2 Materials and methods

### 2.1 Chemicals

QG [flavonoid alcohol extract of marigold (Asteraceae family), with purity ≥80%] was purchased from Chenguang Biotechnology Group Co., Ltd. (Handan, China). The preparation method for QG was reported in reference (Huang et al., 2022). The amount of QG used was accurately calculated, measured, and then thoroughly blended with the premix. The premix was blended with additional components to manufacture rabbit feed. ZEN with a purity guarantee value ≥98% was obtained from Triplebond (Guelph, Canada). ZEN was dissolved in acetic ether and then poured onto talcum powder. The mixture was left in a fume hood overnight to evaporate acetic ether. The dried mixture contained 1,000 mg/kg of ZEN, which was then diluted with toxin-free corn meal to form a premix containing 10 mg/kg of ZEN. The premix was combined with other ingredients to produce rabbit feed.

The basic rabbit diet was formulated according to the feeding standard for rabbits recommended by National Research Council (NRC) (1977) (NRC, 1977). The basic rabbit diet composition and nutrition levels are shown in Table 1.

**TABLE 1:** Composition and nutrient levels of the basal diet (air-dry basis, %).

Ingredients	Content
Corn	8.00
Wheat bran	33.00
Soybean meal	11.00
Wheat middling	4.00
Rice Bran	5.00
Peatnut vite	15.00
Peanut hull	12.50
Artemsia argyi powder	8.00
Limestone	1.05
NaCl	0.50
Ca(HCO_3_)_2_	0.50
Lysine	0.30
Methionine	0.15
Premix^a^	1.00
Total	100.00
Nutritional level^b^	
Digestible energy, MJ/kg	9.33
Crude protein	16.37
Ether extract	3.40
Crude fibre	14.91
Crude ash	12.35
Neutral detergent fibre	42.08
Acid detergent fibre	23.97
Acid detergent lignin	5.62
Calcium	1.60
Total Phosphorus	0.80

^a^ The premix provided the following per kilogram of diet: vitamin A: 10,000 IU; vitamin B_1_: 2 mg; vitamin B_2_: 6 mg; vitamin B_3_: 50 mg; vitamin B_5_: 50 mg; vitamin B_6_: 2 mg; vitamin B_12_: 0.02 mg; vitamin D: 900 IU; vitamin E: 50 mg; vitamin K: 2 mg; choline chloride: 1,000 mg; Biotin: 0.2 mg; Fe: 70 mg; Cu: 20 mg; Zn: 70 mg; Mn: 10 mg; Co: 0.15 mg; I: 0.2 mg; Se: 0.25 mg.

^b^ Nutrient levels were all measured values.

Based on enzyme linked immunosorbent assay (ELISA), the ZEN contents in the formulated diets per group were 80.77, 656.89, and 648.80 μg/kg. The formulated diets contained no other mycotoxins (vomiting toxin, aflatoxin B1, and fumonisin). ZEN, vomiting toxin, and aflatoxin B1 kits were purchased from Shenzhen Finder Biotech. Co., Ltd. (Shenzhen, China), and fumonisin kits were purchased from Enzyme-linked Biotechnology Co., Ltd. (Shanghai, China).

### 2.2 Experimental animals

A total of 90 healthy 41-day-old Hyla rabbits (Feed Resource Development and Evaluation Laboratories, China), weighing 1.41 ± 0.06 kg, with a male-to-female ratio of 1:1, were randomly divided into three groups, including the control group (fed with basic diet), the ZEN addition group (fed with basic diet+ 600 μg/kg ZEN) and the ZEN + QG addition group (fed with basic diet+600 μg/kg ZEN+100 mg/kg QG). Each group had 30 rabbits. The rabbits were fed in single cages (length × width × height: 120 cm × 80 cm × 60 cm) with free access to food and water. The acclimation period lasted 7 days, and the trial period lasted 28 days.

The experimental protocols were approved by the Animal Care and Use Committee of Hebei Agriculture University (Baoding, China) (Protocol: 2023138).

All animal experiments complied with the ARRIVE guidelines were carried out in accordance with the U.K. Animals (Scientific Procedures) Act, 1986 and associated guidelines, EU Directive 2010/63/EU for animal experiments.

### 2.3 Experimental analysis

#### 2.3.1 Growth performance and organ indexes

At the beginning and end of the test, the fasting weight of rabbits in each group was determined as the initial and final weights. In addition, the feed intake of the rabbits in each group during the test was recorded. The average daily gain (ADG) and average daily feed intake (ADFI) were calculated according to the test days. Finally, the gain to feed ratio (F/G) was calculated based on ADG and ADFI.

The live weight of the rabbits was also weighed before slaughter. Subsequently, the weight of each organ was weighed after slaughter to calculate the organ indexes using the following equation: Organ index (g/kg) = organ weight (g)/body weight before slaughter (kg).

#### 2.3.2 Blood and tissue indicators

At the end of the trial period, eight rabbits with weights close to average weight and a male-to-female ratio of 1:1 were selected from each group. After fasting for 12 h, their blood was sampled by cardiac puncture and collected in the vacuum blood collection vessels. The serum was separated by centrifugation for 10 min at 3,000 × g and 4°C and stored at −80°C for subsequent analysis. After blood collection, the rabbits were sacrificed by cervical dislocation. Their liver tissue samples were taken and stored at −80°C for further analysis.

The leves/activities of immunoglobulin A (IgA), immunoglobulin G (IgG), immunoglobulin M (IgM), complement 3 (C3), complement 4 (C4), MDA, tumor necrosis factor-α (TNF-α), interleukin-1β (IL-1β), interleukin-4 (IL-4), interleukin-6 (IL-6), interleukin-10 (IL-10), total antioxidant capacity (TAOC), glutathione peroxidase (GSH-Px), superoxide dismutase (SOD), and catalase (CAT) in serum and liver tissue were detected by ELISA. The TAOC, GSH-Px, CAT, MDA, TNF-α, IL-1β, IL-4, IL-6, and IL-10 kits were procured from Jiangsu Meimian Industrial Co., Ltd (Yancheng, China), while IgA, IgG, and IgM kits were obtained from Nanjing Jiancheng Bioengineering Institute (Nanjing, China). The C3 and C4 kits were procured from Beijing Bo Rui Long Term Technology Co Ltd (Beijing, China). In addition, ALT, AST, ALP, total bile acids (TBA) and total bilirubin (TB) kits were bought from Nanjing Jiancheng Bioengineering Institute (Nanjing, China). The assays were conducted in accordance with the instructions provided by the manufacturer.

#### 2.3.3 Expression of liver-related genes

According to the rabbit gene sequence reported in the GenBank, Primer 6.0 software was employed to design corresponding specific primers, which were synthesized by Sangon Biotech (Shanghai) Co., Ltd. (Shanghai, China) (Table 2).

**TABLE 2:** Sequence of primers for real-time PCR.

Genes	Primer sequence (5'→3′)	Accession no.
*Keap1*	Forward: GGACGGCAACACTGATTC	NM_057152
	Reversed: TCG​TCT​CGA​TCT​GGC​TCA​TA	
*Nrf2*	Forward: CAC​ATC​CAG​ACA​GAC​ACC​AGT	NM_031789
	Reversed: CTA​CAA​ATG​GGA​ATG​TCT​CTG​C	
*H O -1*	Forward: ACA​GGG​TGA​CAG​AAG​AGG​CTA​A	NM_012580
	Reversed: CTG​TGA​GGG​ACT​CTG​GTC​TTT​G	
*NQ O -1*	Forward: CAGCGGCTCCATGTACT	NM_017000
	Reversed: GAC​CTG​GAA​GCC​ACA​GAA​G	
*SOD1*	Forward: GCA​GGC​CCT​CAC​TTT​AAT​CC	NM_001082627.2
	Reversed: CCT​TTG​CCC​AAG​TCG​TCT​TC	
*SOD2*	Forward: TGA​CGG​CTG​TGT​CTG​TTG​GT	L28808.1
	Reversed: GCA​GGT​AGT​AAG​CGT​GTT​CCC	
*GSH-Px*	Forward: GCC​CAG​TCT​GTG​TAC​TCC​TT	NM_001085444.1
	Reversed: CGT​TCT​CCT​GAT​GCC​CAA​AC	
*CAT*	Forward: TGA​CTG​TTG​CTG​GAG​ACT​GG	NM_001076085.1
	Reversed: TGT​GCT​TCT​TCC​TGT​CGA​TG	
*HSP70*	Forward: GAG​TGA​GGA​GAG​GCG​TCA​GT	NC_013671.1
	Reversed: GTT​CTC​ACA​CAG​GTC​GGA​CA	
*TLR4*	Forward: GAG​CAC​CTG​GAC​CTT​TCA​AAT​AAC	NM_001082732
	Reversed: GAA​CTT​CTA​AAC​CAC​TCA​GCC​CTT​G	
*NFkB*	Forward: AAT​GGT​GGA​ATC​TGG​GAA​G	XM_017347386.1
	Reversed: CAA​TGG​CAA​ACT​GTC​TAT​GAA	
*TNF-α*	Forward: CTG​CAC​TTC​AGG​GTG​ATC​G	NM_001082263.1
	Reversed: CTA​CGT​GGG​CTA​GAG​GCT​TG	
*IL-1*β	Forward: CCC​CAA​CCG​TTA​CCC​AAA​GA	NM_001082201.1
	Reversed: GGG​AAC​TGG​GCA​GAC​TCA​AA	
*IL-4*	Forward: CCC​AAG​AAC​ACA​ACC​GAG​AG	NM_001163177.1
	Reversed: AGT​CTG​TCT​GGC​TTC​CTT​CC	
*IL-6*	Forward: TCC​AGG​AGC​CCG​ACT​ATG​AA	NM_001082064.2
	Reversed: TCG​TCA​CTC​CTG​AAC​TTG​GC	
*IL-10*	Forward: AGA​ACC​ACA​GTC​CAG​CCA​TC	NM_001082045.1
	Reversed: GCT​TTG​TAG​ACG​CCT​TCC​TC	
*BAX*	Forward: CCCGCGAGGTCTTTTTCC	XM_008252361.2
	Reversed: CAG​GGC​CTT​GAG​TAC​CAG​CTT	
*Bcl-2*	Forward: GGCTGGGATGCCTTCGT	XM_008261439.2
	Reversed: TTT​CGT​GAA​CTG​TTT​GCA​TAT​CTG	
*Caspase-3*	Forward: GAC​AGT​GGC​ATC​GAG​ACA​GAC​A	NM_008261439.2
	Reversed: GAA​TAG​TAA​CCA​GGT​GCT​GTG​GAA	
*GAPDH*	Forward: TGC​CAC​CCA​CTC​CTC​TAC​CTT​CG	NM_001082253
	Reversed: CGA​AGG​TAG​GGA​TGG​GTG​GCA	

Keap1, kelch-like ECH-associated protein 1; Nrf2, nuclear factor E2-related factor 2; HO-1, heme oxygenase-1; NQO-1, NAD (P) H: quinone oxidoreductase 1; GSH-Px, glutathione peroxidase; SOD1, copper and zinc superoxide dismutase; SOD2, manganese superoxide dismutase; CAT, catalase; HSP70, heat shock protein 70; TLR4, toll-like receptor 4; NFkB, nuclear factor-k-gene binding; TNF-α, tumor necrosis factor-α; IL-1β, interleukin-1β; IL-4, interleukin-4; IL-6, interleukin-6; IL-10, interleukin-10; Bcl-2, B-cell lymphoma/leukemia; Bax, BCL2 Associated X protein; Caspase-3, cysteine-aspartic acid protease-3; GAPDH, glyceraldehyde-3-phosphate dehydrogenase.

The total RNA was extracted from 50 to 100 mg of liver sample using Trizol kit (Invitrogen, Carlsbad, United States) according to the manufacturer’s instructions. The extracted RNA concentration was quantified using NanoDrop Lite (Thermo Fisher, Waltham, United States). Subsequently, cDNA was synthesized by reverse transcription kit (Vazyme, Nanjing, China), with 1 μg of RNA utilized in total following the manufacturer’s instructions. qRT-PCR was performed using the qRT-PCR premix volume consisting of the cDNA template, quantitative primers, and ChamQ Universal SYBR qRTPCR Master Mix kit (Vazyme, Nanjing, China) on the Bio-Rad CFX96 contact real-time PCR detection system (Hercules, United States). The heat-cycling conditions for qRT-PCR were as follows: 95°C for 5 min, followed by 40 cycles at 95°C for 10 s and then at 60°C for 30 s of melt curve analysis. The experiment was replicated thrice with GAPDH as the internal reference gene. The target gene expression was calculated by the 2^-△△Ct^ method.

### 2.4 Statistical analysis

Statistical data analysis was performed using SPSS 20.0 software (IBM-SPSS Inc., Chicago, IL, United States). One-way analysis of variance (ANOVA) was utilized to assess significant differences between groups, while the Duncan method was employed for multiple comparisons. A *p*-value less than 0.05 (*p* < 0.05) indicated statistical significance between groups.

## 3 Results

### 3.1 Growth performance

There were no significant differences in ADG, ADFI, and F/G of rabbits among the three groups at *p* < 0.05 (Table 3). Rabbits in the ZEN group had a 0.07 percent increase in ADG compared to the control group, while those in the ZEN + QG group had a 4.32 percent decrease (*p* > 0.05). Rabbits in the ZEN group showed a 1.72 percent increase in ADFI compared to controls, while rabbits in the ZEN + QG group showed a 7.82 percent decrease in ADFI (*p* > 0.05).

**TABLE 3:** Effect of QG on growth performance of rabbits fed a ZEN-contaminated diet.

Items	Control group	ZEN group	ZEN + QG group	SEM	*p*-value
ADG, g	41.68	41.71	39.88	1.218	0.823
ADFI, g	173.43	176.41	159.86	3.696	0.199
F/G	4.27	4.35	4.06	0.089	0.444

Values are means (*n* = 30).

ZEN, group, 600 μg/kg zearalenone supplementation group; ZEN + QG, group, 600 μg/kg zearalenone and 100 mg/kg quercetagetin supplementation group; SEM, the standard error of the means; ADG, average daily gain; ADFI, average daily feed intake; F/G, gain to feed ratio.

### 3.2 Organ indexes

The sacculus rotundus index of rabbits in the ZEN + QG group was significantly lower than the control group (*p* < 0.05) (Table 4). However, there were no significant differences in the liver, kidney, and spleen indexes of rabbits among the three study groups (*p* > 0.05).

**TABLE 4:** Effect of QG on organ indexes of rabbits fed a ZEN-contaminated diet.

Items	Control group	ZEN group	ZEN + QG group	SEM	*p*-value
Liver, g/kg	32.87	33.49	34.59	0.905	0.751
Kidney, g/kg	6.39	6.66	7.23	0.203	0.232
Spleen, g/kg	0.68	0.87	0.67	0.068	0.431
Round vesicle, g/kg	1.16^b^	0.98^ab^	0.88^a^	0.049	0.044

a, b, c Within a row, means with different superscripts differ significantly (*p* < 0.05). Values are means (*n* = 8).

ZEN, group, 600 μg/kg zearalenone supplementation group; ZEN + QG, group, 600 μg/kg zearalenone and 100 mg/kg quercetagetin supplementation group; SEM, the standard error of the means.

### 3.3 Serum biochemical and immune indicators

Compared to the control group, the ALT and ALP activities and the TBA and TB levels in the blood of rabbits in the ZEN group were significantly increased, while the IgA and C3 levels were significantly decreased (*p* < 0.05). On the contrary, there were no significant differences in these indexes between the ZEN + QG group and the control group (*p* > 0.05), except for the TBA level, which was significantly higher than in the control group (*p* < 0.05). Compared to the ZEN group, the ALT activity and TBA level in the blood of rabbits in the ZEN + QG group were significantly decreased (*p* < 0.05). However, no significant differences in blood AST activity and the contents of IgG, IgM, and C4 were observed among the three study groups (Table 5).

**TABLE 5:** Effect of QG on serum biochemical and immune indicators of rabbits fed a ZEN-contaminated diet.

Items	Control group	ZEN group	ZEN + QG group	SEM	*p*-value
ALT, U/L	19.54^a^	29.38^b^	23.18^a^	1.264	0.002
AST, U/L	15.36	19.81	17.75	0.790	0.064
ALP, U/L	83.27^a^	96.33^b^	86.04^ab^	2.290	0.045
TBA, μmol/L	5.73^a^	10.15^b^	7.65^c^	0.469	<0.001
TB, μmol/L	21.80^a^	32.59^b^	27.86^ab^	1.380	0.002
IgG, mg/L	875.87	791.21	885.90	20.286	0.109
IgA, mg/L	65.20^b^	53.15^a^	59.53^ab^	1.934	0.031
IgM, mg/L	16.80	14.18	17.57	0.863	0.254
C3, μg/mL	112.62^b^	90.34^a^	101.48^ab^	3.742	0.044
C4, μg/mL	147.68	138.02	152.23	4.192	0.385

a, b, c Within a row, means with different superscripts differ significantly (*p* < 0.05). Values are means (*n* = 8).

ZEN, group, 600 μg/kg zearalenone supplementation group; ZEN + QG, group, 600 μg/kg zearalenone and 100 mg/kg quercetagetin supplementation group; SEM, the standard error of the means; ALT, alanine aminotransferase; AST, aspartate aminotransferase; ALP, alkaline phosphatase; TBA, total bile acid; TB, total bilirubin; IgG, immunoglobulin G; IgA, immunoglobulin A; IgM, immunoglobulin M; C3, complement 3; C4, complement 4.

### 3.4 Serum and liver antioxidant indexes

Compared to the control group, GSH-Px and SOD activities in the blood of rabbits in the ZEN group were significantly decreased, while the MDA level was significantly increased (*p* < 0.05) (Table 6). However, they were not different from the ZEN + QG group (*p* > 0.05). Additionally, the TAOC and GSH-Px activities in the liver of rabbits in the ZEN group were significantly lower (*p* < 0.05), and the MDA level was significantly higher (*p* < 0.05) than the control group. However, the ZEN + QG group showed no significant differences in these indexes (*p* > 0.05). Besides, there was no significant difference in the serum and liver CAT activity of rabbits in each group.

**TABLE 6:** Effect of QG on serum and liver antioxidant indexes of rabbits fed a ZEN-contaminated diet.

Items	Control group	ZEN group	ZEN + QG group	SEM	*p*-value
Serum					
TAOC, U/mL	32.06	28.43	34.48	1.529	0.277
GSH-Px, U/L	162.71^b^	132.69^a^	154.90^b^	5.106	0.037
SOD, U/mL	13.83^b^	10.61^a^	11.38^ab^	0.541	0.032
CAT, U/mL	24.86	22.18	26.13	0.889	0.183
MDA, nmol/L	16.68^a^	23.00^b^	19.26^ab^	0.994	0.025
Liver					
TAOC, U/mg prot	20.22^b^	13.24^a^	17.91^ab^	1.173	0.039
GSH-Px, U/mg prot	185.17^b^	131.61^a^	165.51^b^	6.616	0.001
SOD, U/mg prot	15.33	14.12	17.39	0.714	0.169
CAT, U/mg prot	30.24	34.69	36.78	1.454	0.174
MDA, U/mg prot	30.69^a^	41.34^b^	32.14^a^	1.695	0.013

a, b, c Within a row, means with different superscripts differ significantly (*p* < 0.05). Values are means (n = 8).

ZEN, group, 600 μg/kg zearalenone supplementation group; ZEN + QG, group, 600 μg/kg zearalenone and 100 mg/kg quercetagetin supplementation group; SEM, the standard error of the means; TAOC, total antioxidant capacity; GSH-Px, glutathione peroxidase; SOD, superoxide dismutase; CAT, catalase; MDA, malondialdehyde.

### 3.5 Serum and liver inflammation indicators

Compared to the control group, the IL-4 level in the blood and the TNF-α and IL-4 levels in the liver of rabbits in the ZEN group were significantly increased, while the IL-10 level in the liver were significantly reduced (*p* < 0.05) (Table 7). IL-4 level in the blood and liver of rabbits in the ZEN + QG group was also significantly higher than in the control group (*p* < 0.05), while the IL-4 level in the liver was significantly lower than that in the ZEN group (*p* < 0.05). There were no significant differences in IL-1β and IL-6 levels in rabbits across the three study groups (*p* > 0.05).

**TABLE 7:** Effect of QG on serum and liver inflammation indicators of rabbits fed a ZEN-contaminated diet.

Items	Control group	ZEN group	ZEN + QG group	SEM	*p*-value
Serum					
TNF-α, pg/mL	437.40	479.75	430.65	10.293	0.104
IL-1β, pg/mL	83.53	91.26	84.70	2.140	0.295
IL-4, pg/mL	47.37^a^	62.76^b^	60.42^b^	2.472	0.016
IL-6, pg/mL	195.00	231.97	215.47	7.300	0.114
IL-10, pg/mL	619.53	637.28	651.16	12.170	0.589
Liver					
TNF-α, pg/mg prot	313.76^a^	376.53^b^	333.57^ab^	10.691	0.042
IL-1β, pg/mg prot	62.67	69.38	61.36	2.007	0.223
IL-4, pg/mg prot	34.40^a^	64.45^c^	56.75^b^	2.920	<0.001
IL-6, pg/mg prot	121.71	142.22	135.98	4.303	0.403
IL-10, pg/mg prot	560.62^b^	478.11^a^	526.90^ab^	12.667	0.020

a, b, c Within a row, means with different superscripts differ significantly (*p* < 0.05). Values are means (n = 8).

ZEN, group, 600 μg/kg zearalenone supplementation group; ZEN + QG, group, 600 μg/kg zearalenone and 100 mg/kg quercetagetin supplementation group; SEM, the standard error of the means; TNF-α, tumor necrosis factor-α; IL-1β, interleukin-1β; IL-4, interleukin-4; IL-6, interleukin-6; IL-10, interleukin-10.

### 3.6 Expression level of liver-related genes

#### 3.6.1 Expression level of antioxidant-related genes in the liver

**FIGURE 1:**
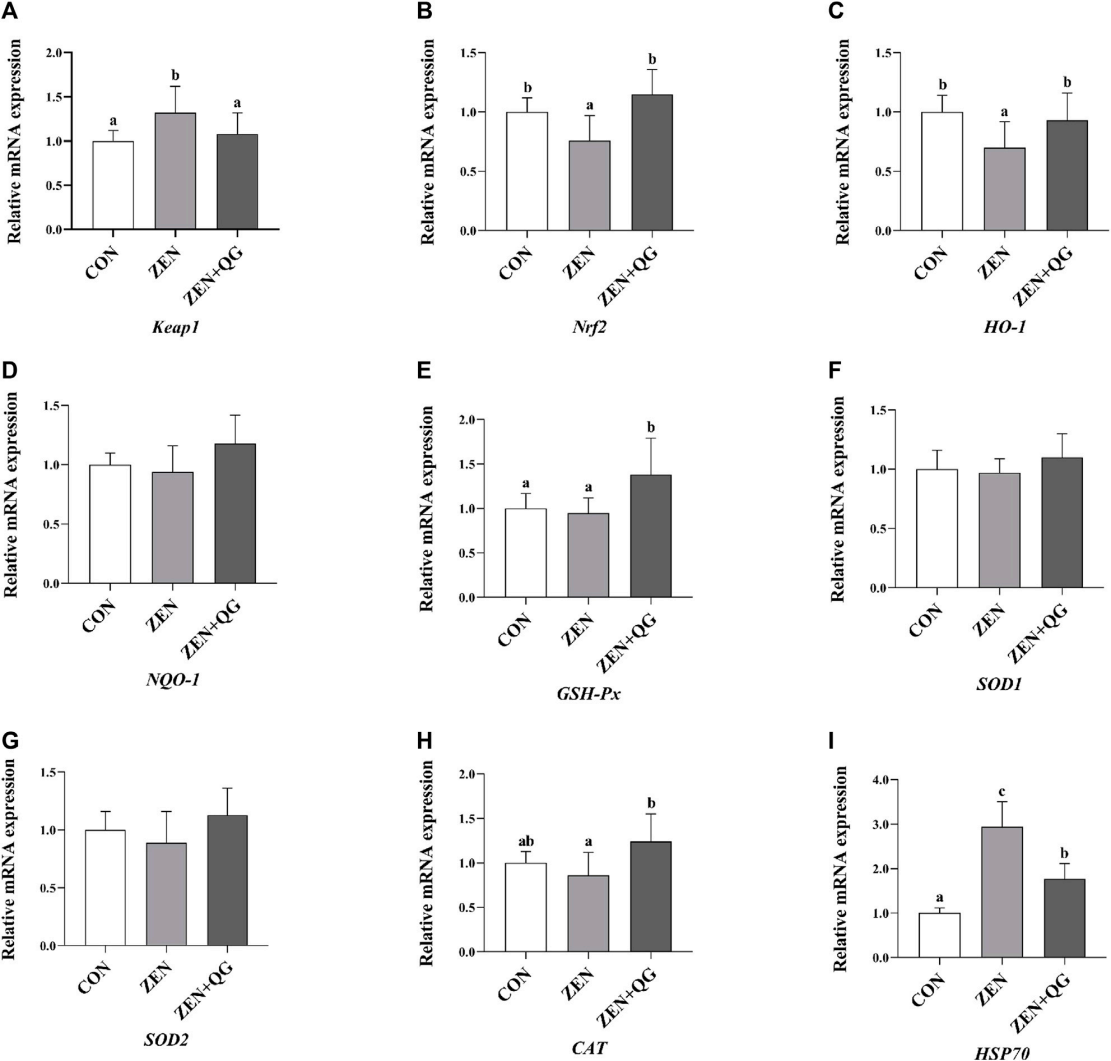
Effects of QG on the expression levels of liver antioxidant-related genes in rabbits fed on a ZEN-contaminated diet. The mRNA level of genes were determined by qRT-PCR. Bars represent the means ± standard deviation (SD) (*n* = 8). Above the bar no letter or the same letter mean no significant (*p* > 0.05), while with different letter mean significant difference (*p* < 0.05). Kelch-like ECH-associated protein 1 (*Keap1*) **(A)**, Nuclear factor E2 related factor 2 (*Nrf2*) **(B)**, Heme oxygenase-1 (*HO-1*) **(C)**, NAD(P)H: quinone oxidoreductase 1 (*NQO-1*) **(D)**, Glutathione peroxidase (*GSH-Px*) **(E)**, Copper and zinc superoxide dismutase (*SOD1*) **(F)**, Manganese superoxide dismutase (*SOD2*) **(G)**, Catalase (*CAT*) **(H)**, Heat shock protein 70 (*HSP70*) **(I)**.

Compared to the control group, the abundance of *Keap1* and *HSP70* mRNAs in the livers of rabbits in the ZEN group were significantly increased, while the *Nrf2* and *H O -1* mRNAs were significantly decreased (*p* < 0.05) (Figure 1). In the ZEN + QG group, the abundance of *GSH-Px* and *HSP70* mRNAs in the liver significantly increased compared to the control (*p* < 0.05). Compared to the ZEN group, the abundance of *Nrf2*, *H O -1*, *GSH-Px*, and *CAT* mRNAs in the liver of rabbits in the ZEN + QG group was significantly increased (*p* < 0.05), while *Keap-1* and *HSP70* mRNAs were significantly decreased (*p* < 0.05).

#### 3.6.2 Expression level of genes related to the anti-inflammatory reaction in the liver

**FIGURE 2:**
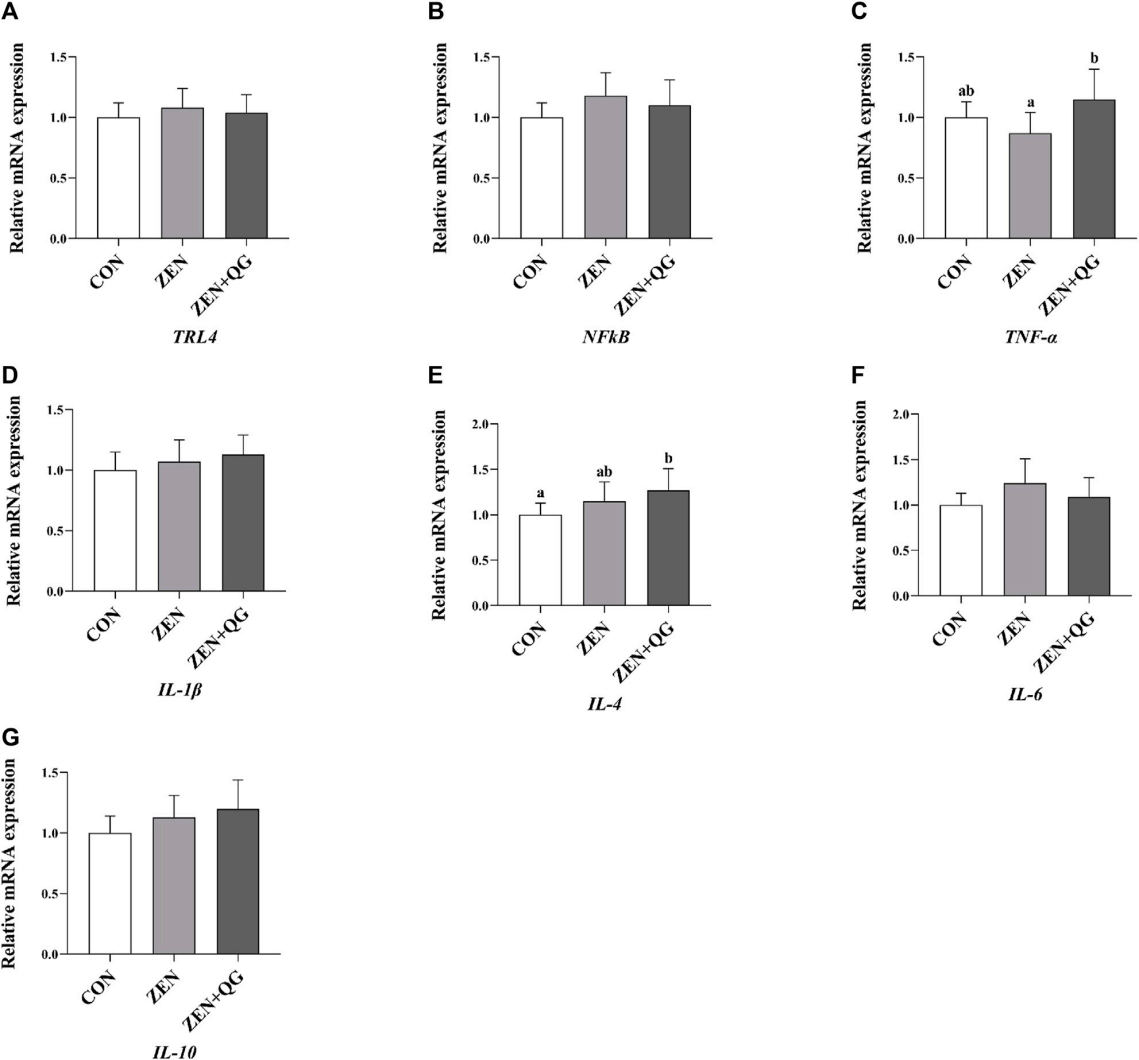
Effects of QG on the expression levels of liver anti-inflammatory reaction genes in rabbits fed on a ZEN-contaminated diet. The mRNA level of genes were determined by qRT-PCR. Bars represent the means ± standard deviation (SD) (*n* = 8). Above the bar no letter or the same letter mean no significant (*p* > 0.05), while with different letter mean significant difference (*p* < 0.05). Toll-like receptor 4 (*TLR4*) **(A)**, Nuclear factor-k-gene binding (*NFkB*) **(B)**, Tumor necrosis factor-α (*TNF-α*) **(C)**, Interleukin-1β (*IL-1*β) **(D)**, Interleukin-4 (*IL-4*) **(E)**, Interleukin-6 (*IL-6*) **(F)**, Interleukin-10 (*IL-10*) **(G)**.

Compared to the ZEN + QG group, the abundance of *IL-4* mRNA in the liver of rabbits in the control group and *TNF-α* mRNA in the liver of rabbits in the ZEN group were significantly decreased (*p* < 0.05) (Figure 2).

#### 3.6.3 Expression level of genes related to anti-apoptosis in the liver

**FIGURE 3:**
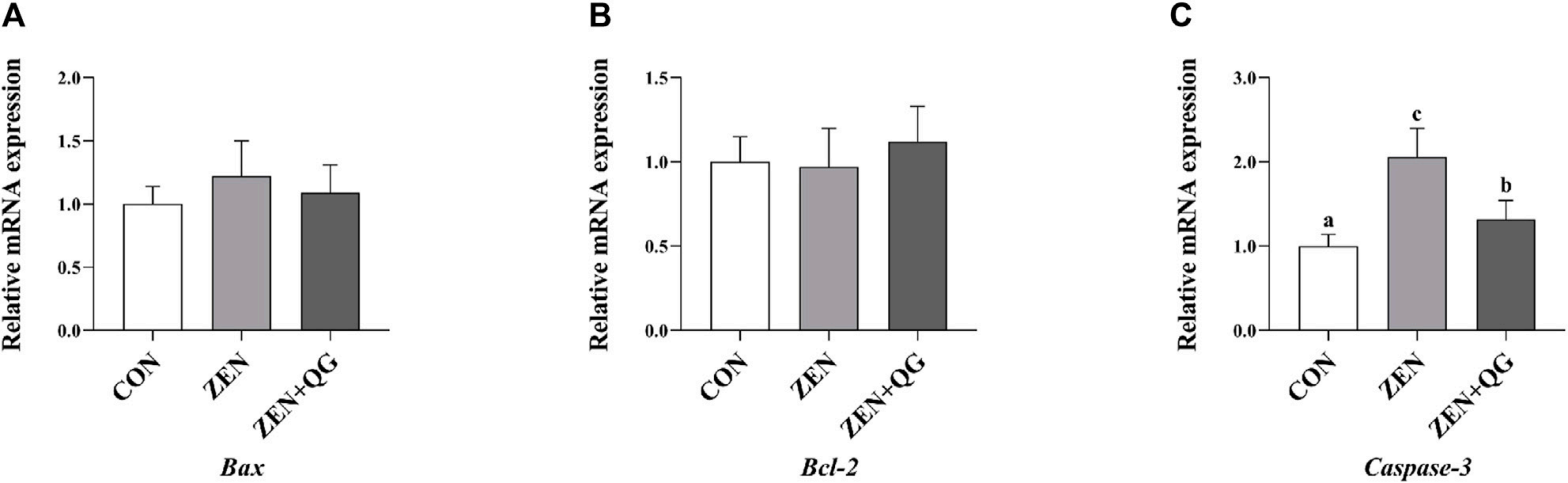
Effects of QG on the expression levels of liver anti-apoptosis genes in rabbits fed on a ZEN-contaminated diet. The mRNA level of genes were determined by qRT-PCR. Bars represent the means ± standard deviation (SD) (*n* = 8). Above the bar no letter or the same letter mean no significant (*p* > 0.05), while with different letter mean significant difference (*p* < 0.05). B-cell lymphoma/leukemia (*Bcl-2*) **(A)**, BCL2 Associated X protein (*Bax*) **(B)**, Cysteine-aspartic acid protease-3 (*Caspase-3*) **(C)**.

Compared to the control group, the abundance of *Caspase-3* mRNA in the liver of rabbits in ZEN and ZEN + QG groups was significantly increased (*p* < 0.05). Furthermore, its abundance in the ZEN group was significantly higher than that in the ZEN + QG group (*p* < 0.05) (Figure 3).

## 4 Discussion

There are few reports on the effect of QG on animals’ growth performance. This study reports the effect of QG on rabbits for the first time. In this study, there were no significant differences in ADG, ADFI, F/G, and the liver, kidney, and spleen organ indexes among the rabbits across the three groups, implying that the dosage and time of ZEN and QG administration had no significant effect on the growth performance of rabbits, including their liver, kidney, and spleen development. This is partially consistent with a previous report where QG (3.2, 4.8, or 6.4 mg/kg, fed for 42 days) had no significant effect on the spleen, thymus, and bursa of fabricius organ indexes in broilers (Wu et al., 2022b). ZEN effects on growth performance are related to dosage, exposure time, animal species, growth stage, and other factors (Gajęcka et al., 2016; Chen et al., 2019; Liu et al., 2020; Ropejko and Twarużek, 2021). In ducks, ZEN administration (5 mg/kg, fed for 35 days) has no significant effect on ADG, total feed intake, feed conversion rate, and the liver, spleen, and kidney weights (Peillod et al., 2021). Similarly, ZEN (0.8 mg/kg, fed for 28 days) has no significant effect on ADG, ADFI, feed conversion rate, and the liver and kidney-to-body weight ratio of growing pigs (Reddy et al., 2018). This is partially consistent with the findings in this study. Herein, the sacculus rotundus index in the ZEN + QG group was significantly lower than the control group, which may be related to the interaction between ZEN enterotoxicity and QG biological activity.

The liver is the main organ for ZEN metabolism in rabbits and a target organ for ZEN toxicity (Kuiper-Goodman et al., 1987). ZEN (50 μM/kg BW, oral intake for 49 days) administration induces rabbit hepatocyte injury, liver dysfunction, and inflammatory reaction, significantly increasing the AST activity, TB level, and the number of white blood cells, monocytes, and eosinophils (Tsouloufi et al., 2019). It also significantly decreases urea, creatinine, glucose, total calcium, sodium, and potassium concentrations (Tsouloufi et al., 2019). In this experiment, ZEN exposure significantly increased the indicators for liver injury, such as the blood ALT and ALP activities, TBA and TB levels, and the liver *Caspase-3* mRNA abundance in rabbits, compared to the control group, implying that ZEN exposure induced liver injury in rabbits. However, adding QG alleviated the liver injury caused by ZEN exposure. In the ZEN + QG group, except for the TBA level and *Caspase-3* mRNA abundance, which were significantly lower than in the ZEN group, though still significantly higher than in the control group, the other indicators were restored to the level in the control group. There are several reasons for that. On the one hand, oxidative stress is an important pathway for ZEN to induce hepatotoxicity (Tatay et al., 2017). Besides, QG not only has a strong capacity to scavenge DPPH, ABTS, ·OH and other free radicals (Fuentes et al., 2021) but also enhances the antioxidant capacity in the liver through the Nrf2/ARE signaling pathway (Wu et al., 2022b). Therefore, in this study, supplementing the diet with QG alleviated the oxidative damage to the liver of rabbits in the ZEN + QG group. On the other hand, the inflammatory reaction is critical for ZEN to induce hepatotoxicity, and QG has anti-inflammatory activity. QG has a strong inhibitory effect on the inflammatory response of Caco-2 cells induced by silver nanoparticles (Rufino et al., 2021). Besides, QG has antiviral activity, which positively maintains liver health and stability (Seyedi et al., 2016). Therefore, QG alleviated the reduction of IgA and C3 levels induced by ZEN, given its antioxidant activity, which enhanced the anti-stress ability of immune cells. Similarly, QG alleviated the impact of oxidative stress on human lymphoblasts by eliminating free radicals and improving the activity of antioxidant enzymes (Chkhikvishvili et al., 2016).

Moreover, ZEN exposure reduces the activity of antioxidant enzymes. It stimulates electrons to leak out of the respiratory chain, generating excessive free radicals, which imbalance the animal oxidation and antioxidant systems, leading to oxidative stress and damage (Ren et al., 2016; Tatay et al., 2017; Zheng et al., 2019). For example, ZEN (0.5, 1.0, and 1.5 mg/kg, fed for 35 days) significantly reduces the GSH-Px and SOD activities, which significantly increase the MDA level and jejunal oxidative stress by regulating the Keap1-Nrf2 signal pathway (Cheng et al., 2019). In this study, ZEN exposure significantly reduced the activity of certain antioxidant enzymes in the blood and liver of rabbits in the ZEN group. It significantly increased the MDA level and *HSP70* mRNA abundance, implying that it induced oxidative stress in the liver. However, QG supplementation restored the antioxidant enzyme activity and the MDA level in the blood and liver of rabbits in the ZEN + QG group to the levels in the control group, thereby alleviating the effect of ZEN exposure on the liver of rabbits. This is related to the antioxidant activity of QG. Quercetin has several biological activities, including antioxidant activity (Boots et al., 2008). Its antioxidant activity is associated with the catechol group on the B ring and the hydroxyl group on site 3 of the A and C rings (Boots et al., 2008). QG and quercetin are flavonoid alcohols. Regarding structure, QG has one more phenolic hydroxyl group at position 6 of ring A than quercetin; thus, theoretically, QG has a stronger biological activity (Xu et al., 2011). In addition, the oxygen atom of QG on the 4-position of the carbonyl group has a stronger coordination ability, and its polyhydroxy space structure is conducive to the formation of metal complexes with diverse structures, which is an important source of various QG biological activities, including the antioxidant activity (Xu et al., 2011). Besides, QG has a similar scavenging ability as quercetin towards 2,2′-diazobium (3-ethylbenzothiazoline-6-sulfonic acid) and 2,2-biphenyl-1-picrylhydrazine, with IC50 values of 12.16 and 12.38 μmol/L, 27.12 and 27.85 μmol/L, respectively. Notably, QG (6.4 mg/kg, fed for 42 days) significantly increased the blood GSH-Px activity and the liver GSH-Px, and SOD activities in broilers. At the same time, QG significantly reduces the MDA level, which is partially consistent with the results in this study (Wu et al., 2022b). Besides, QG supplementation significantly reduced the abundance of *Keap1* mRNA in the liver of rabbits in the ZEN + QG group compared to the ZEN group. Meanwhile, the abundance of *Nrf2*, *HO -1*, *GSH-Px*, and *CAT* mRNA was significantly higher in the ZEN + QG group than in the ZEN group, implying that QG alleviated oxidative damage caused by ZEN exposure through the Keap1-mediated Nrf2-ARE signal pathway. This is consistent with the results by Wu et al. (Wu et al., 2022b) on the effects of QG on broilers. Nrf2 is a key transcription factor participating in many processes, such as cell antioxidation, anti-inflammatory response, and detoxification, which plays a regulatory role in the redox homeostasis regulation of cells in an independent or dependent manner on the Keap1 pathway (Bryan et al., 2013).

ZEN exposure induced a significant increase in the IL-4 level in the blood, IL-4 and TNF-α levels in the liver, and a significant decrease in the IL-10 level in the ZEN group, implying that it caused some inflammation in the liver. ZEN induces an inflammatory reaction and promotes the production of TNF-α through the c-Jun amino-terminal kinase signal pathway (Pistol et al., 2015). ZEN also enhances the production of IL-4 by stimulating Th1 and Th2 lymphocytes (Obremski, 2014). Additionally, ZEN inhibits the production of IL-10 through the p-38MAPK signaling pathway (Pistol et al., 2014). The addition of QG in the rabbit diet reversed the increase of IL-4 and TNF-α levels and the decrease in IL-10 levels induced by ZEN, thereby exerting an alleviative role. This is possible because free radicals are the effectors of inflammatory reactions, and excessive oxygen free radicals induce the inflammatory reaction in the body through model or non-model receptors (Gill et al., 2010). QG alleviates the inflammatory reaction caused by ZEN exposure by inhibiting the production of free radicals. In addition, QG promotes transforming growth factor β1 expression in HaCaT keratinocytes, which regulates inflammation and immune responses (Kang et al., 2014).

## 5 Conclusion

QG alleviates the oxidative damage induced by ZEN and the liver injury caused by inflammatory reaction through the Keap1-Nrf2-ARE signal pathway, thereby protecting the liver.

## Data Availability

The original contributions presented in the study are included in the article/Supplementary material, further inquiries can be directed to the corresponding author.
